# Simultaneous sequestration of heavy metals and organic pollutants from vehicles industrial wastewater via iron-based electrocoagulation: optimization of operating dynamics and environmental compliance

**DOI:** 10.1038/s41598-026-60001-2

**Published:** 2026-07-04

**Authors:** Omar Bahaa Eldin Abdel Wahab, Enas Abou-Taleb, Usama Fathy Mahmoud, Mohamed Saad Hellal

**Affiliations:** 1 Civil Engineering Department, Faculty of Engineering, El Azhar University, Cairo, Egypt; 2 Water Pollution Research Department, National Research Centre, El Behooth St., Dokki, P.O. Box 12622, Cairo, Egypt

**Keywords:** Electrocoagulation, Automotive wastewater, Nickel, Hexavalent chromium Cr(VI), Iron electrode, Wastewater treatment, Chemistry, Engineering, Environmental sciences

## Abstract

This study investigates the simultaneous removal of Nickel (Ni^+2^), hexavalent chromium Cr(VI), and Chemical Oxygen Demand (COD) from automotive industrial wastewater using an advanced electrocoagulation (EC) process. A key novelty of this work lies in the development of a distinct, optimized iron electrode configuration represented by circular iron electrodes instead of square or rectangular, integrated with optimum operation criterions to maximize pollutant mitigation while minimizing resource consumption. Batch experiments were conducted using iron electrodes on real effluent characterized by baseline concentrations of 12 ± 0.40, 11 ± 0.30 and 2500 ± 75 mg L^− 1^ for Ni^+2^, Cr(VI) and COD respectively. Through systematic optimization, the critical design criteria for a scalable system were established at an initial pH of 7, a current density (CD) of 3 mA cm^− 2^ and an electrolysis time of 40 min. These optimal parameters successfully balanced peak treatment efficiency with the lowest possible electrode consumption and electrical energy demand. The residual Ni^+2^, Cr, COD was 0.096 ± 0.02, 0.025 ± 0.01 and 940 ± 15 mg L^− 1^ respectively, while energy consumption reached 1.65 kWh m^− 3^ and the electrode consumption was recorded at 0.60 kg m^− 3^. Transitioning from batch to a continuous-flow regime under optimal batch operating conditions yielded residual concentrations of Ni^+2^, Cr and COD was 0.2 ± 0.02, 0.15 ± 0.01 and 915 ± 15 mg L^− 1^ respectively, achieving full compliance with stringent national environmental discharge regulations. Techno-economic analysis demonstrated a highly competitive operational cost 0.36 USD m^− 3^. Solid-state sludge characterization via SEM, EDX, XRD, and FTIR confirmed the stable immobilization and encapsulation of heavy metals within a robust iron-rich crystalline matrix. Ultimately, the novel electrode geometry and derived design framework provide a technically viable, economically sustainable, and scalable solution for automotive wastewater remediation.

## Introduction

Heavy metals and organic contaminants are types of harmful pollutants commonly found in significant amounts inside industrial wastewater. The lack of rules controlling the discharge of hazardous chemicals into the environment degrades water quality and negatively impacts aquatic ecosystems^1^. These contaminants provide significant threats to human health via bioaccumulation, result in a substantial decline in aquatic biodiversity, and undermine the resilience of aquatic ecosystems^2^. Automotive wastewater is distinguished by elevated concentrations of organic matter, quantified as COD, derived from oils, greases, and detergents utilized in manufacturing, cleaning operations, painting, and surface treatment activities^3^. The effluents of automotive wastewater contain toxic heavy metals such as (Ni²⁺), trivalent Chromium Cr(III) and Cr(VI), along with others like Zinc (Zn), Aluminum (Al), Phosphorus (P), and Manganese (Mn), primarily from plating and surface treatment activities^4^. Oil and grease are major contaminants resulting from engine washing and mechanical component degreasing, forming a barrier on the water surface^4^. High levels of turbidity are caused by suspended particles, dirt, and colloidal matter washed off from automotive during the cleaning process^5^. The efficient biological treatment of the wastewater of the automotive industry is difficult, mainly because of the low (BOD/COD) ratio. Moreover, wastewater of the automotive industry contains heavy metals and other chemical substances that may have toxic effects^6^.

Ni^+2^ over its critical threshold may result in significant pulmonary and renal complications, in addition to gastrointestinal discomfort, pulmonary fibrosis, and dermal dermatitis^7^. Cr exists in aquatic environments primarily in two forms: Cr(III) and Cr(VI). Generally, Cr(VI) is more harmful than Cr(III). Cr(VI)impacts human physiology, bio accumulates in the food chain, and induces significant health issues, from mild dermal irritation to lung cancer^8^. Numerous approaches including physical, chemical, and biological processes such as adsorption, bio sorption, precipitation, ion exchange, reverse osmosis, filtration, and other membrane separations, are utilized to treat wastewaters^9^. All of these techniques have disadvantages. For example, the adsorption process requires a long treatment time and ad sorbent pre-treatment. Large quantities of sludge are produced by precipitation, chemical treatment, and coagulation processes, which causes environmental problems. The ion-exchange processes considered expensive and has high maintenance costs^10^. In contrast to traditional chemical coagulation, EC diminishes chemical use, enhances removal efficacy, and streamlines operation, rendering it a more sustainable and efficient approach for intricate industrial effluents^11^.

Upon the application of direct current in EC, the anode disintegrates, releasing metal ions that undergo hydrolysis to generate metal hydroxides and poly-hydroxide complexes, which function as in situ coagulants and destabilize contaminants^11^. These hydroxide species neutralize charge and facilitate the aggregation of dissolved and colloidal pollutants into flocs, which can subsequently be eliminated through sedimentation or flotation^12^. EC is most effective when the CD, pH, electrode type, electrode spacing, electrolysis duration, and conductivity are optimized^13^. All of these factors influence the development of flocs, the production of coagulants, and the degradation of pollutants^14^.The flocs generated by EC are generally large, containing less bound water and exhibiting more stability, thereby facilitating their removal through filtration. EC requires uncomplicated equipment and can be tailored for any capacity of wastewater treatment facility^15^. This approach eliminates the need for chemical additives, hence minimizing the potential for secondary pollutant creation. It requires less current and can consequently be powered by sustainable methods, including solar energy, wind turbines, and fuel cells^16^.

Electrocoagulation using iron electrodes has advantages over electrocoagulation using other electrodes, especially in removing heavy metals such as Cr(VI). Removal of Cr(VI) via EC involves a multi-stage electrochemical pathway. Primarily, Cr(VI) is reduced to Cr(III) by the ferrous ions Fe^+2^ generated at the sacrificial iron anode, which act as the essential reducing agent in the process. Subsequently, the resulting Cr(III) species are removed through precipitation as insoluble chromium hydroxide [Cr(OH)_3_] or via adsorption onto the surface of the concurrently formed iron hydroxide flocs [Fe(OH)_n_]^17^. Also Electrocoagulation using iron electrodes is bather than other electrodes for removal Ni^+2^. Iron works efficiently at an alkaline pH, and therefore Ni^+2^ removal occurs through a combined combination of chemical precipitation of Ni^+2^ hydroxide and adsorption on the surface of iron hydroxides^18^. Electrocoagulation via iron has been thoroughly examined for the treatment of diverse forms of wastewater, including urban wastewater^19^, petrochemical effluent^20^, nutritional and protein wastewater^21^.

Numerous researchers have examined the extraction of Ni^+2^, Cr (VI), and COD from industrial wastewater by electrocoagulation via iron under varying operational parameters. Verma et al.^17^ demonstrated that electrocoagulation using iron electrodes achieved 100% removal efficiency for both Cr(III) and Cr(VI). The process reached completion within 45 min at an optimal CD of 50 mA cm^-2^ and a pH of 4. Zaroual et al.^13^ studied the use of iron electrodes in an electrocoagulation technique to treat textile effluent, just three min of electrolysis enough at a potential of 600 mV to remove 84% of the original COD of 485 mgL^-1^. Occasionally, it is essential to integrate electrocoagulation with additional systems, such as electro-oxidation, to eliminate pollutants, particularly those related COD and colors. Gökkuş et al.^22^ investigated a sequential electrochemical process to treat Acid Brown 14 diazo dye using a continuous flow EC reactor with Fe electrodes, operated at a current of 13.70 A within a pH range of 4.0–10.0 for a treatment time of 18 min. While EC achieved complete decolorization, mineralization was minimal. To enhance treatment, the effluent was subjected to advanced oxidation; the sequential EC–Electro-Fenton (EC-EF) process achieved 68% TOC removal, while the sequential EC–Photo electro Fenton (EC-PEF) process utilizing a Pt anode, an air-diffusion cathode, and a UVA lamp at 50 mA cm^-2^ and pH 3.0 significantly increased mineralization to 90% in chloride and 97% in sulfate media. Nidheesh and Gökkuş.^23^ appraised the aerated iron electrocoagulation process as an innovative and extremely effective technique for water and wastewater treatment. Prior to change, pollutant removal in traditional electrocoagulation predominantly depends on established coagulation mechanisms. Following process optimization by aeration and the substitution of conventional iron cathodes with non-corrosive, carbon-based materials (such as graphite or carbon nanotubes), the system advances to a peroxi-coagulation phase. This improvement markedly improves efficiency by producing potent advanced oxidants such as hydrogen peroxide (H_2_O_2_), hydroxyl radicals (OH*), and ferryl ions to efficiently oxidize recalcitrant organic molecules and arsenite. Yıldız et al.^24^ demonstrated that the peroxi-coagulation process using an iron wire anode and a carbon fiber cathode achieved 100% turbidity removal and 69.8% total organic carbon (TOC) removal efficiencies for real textile wastewater. The process reached optimal performance in an oxygenated medium under a constant applied current of 100 mA, a wastewater dilution rate of C_0_/4, and an air flow rate of 0.1 L min^-1^. Consequently, this hybrid approach systematically improves pollutant degradation efficiency while lowering overall energy consumption and operational costs compared to traditional, non-aerated configurations. Many gaps in the majority of earlier studies that use electrocoagulation since the best type of electrodes for removing pollutants was not chosen, and the economic factor was not taken into account while creating the required treatment model. The purpose of this study is to assess the technical and financial viability of treating wastewater from automotive manufacturing facilities using electrocoagulation with iron. This wastewater contains Ni^+2^, Cr(VI), and a high COD. In order to guarantee the removal of pollutants while simultaneously lowering electricity and electrode consumption, the novelty model was constructed differently, concentrating on the geometry of the iron electrodes. In order to achieve residual pollution levels that comply with environmental requirements, the ideal operational parameters for wastewater treatment, such as electrolysis time, pH, and CD, were investigated.

## Materials and methods

### Source of wastewater

Industrial wastewater is collected from vehicle manufacturing plant, with an estimated output of approximately 150–200 m^3^d^− 1^. This water results from surface treatment and cleaning methods designed to remove impurities, as well as from painting activities. Table 1 presents the parameters of industrial wastewater utilized in this study.

**Table 1:** The Characteristics of raw wastewater from vehicles industry.

Parameters	Unit	Value
pH	--	8–9
Chemical Oxygen Demand (COD)	mg L^− 1^	2500 ± 75
Soluble Chemical Oxygen Demand (sCOD)	mg L^− 1^	1100 ± 250
Biochemical Oxygen Demand (BOD)	mg L^− 1^	1150 ± 150
Total Suspended Solids (TSS)	mg L^− 1^	500 ± 150
Total Dissolved Solids (TDS)	mg L^− 1^	3280 ± 400
Total Kjeldahl Nitrogen (TKN)	mg L^− 1^	17 ± 7
Total Phosphorus (TP)	mg L^− 1^	25 ± 5
Oil and grease	mg L^− 1^	160 ± 25
Nickel (Ni^+2^)	mg L^− 1^	12 ± 0.40
Chromium Cr (VI)	mg L^− 1^	11 ± 0.30

### Design and operation of EC reactor

**Fig. 1:**
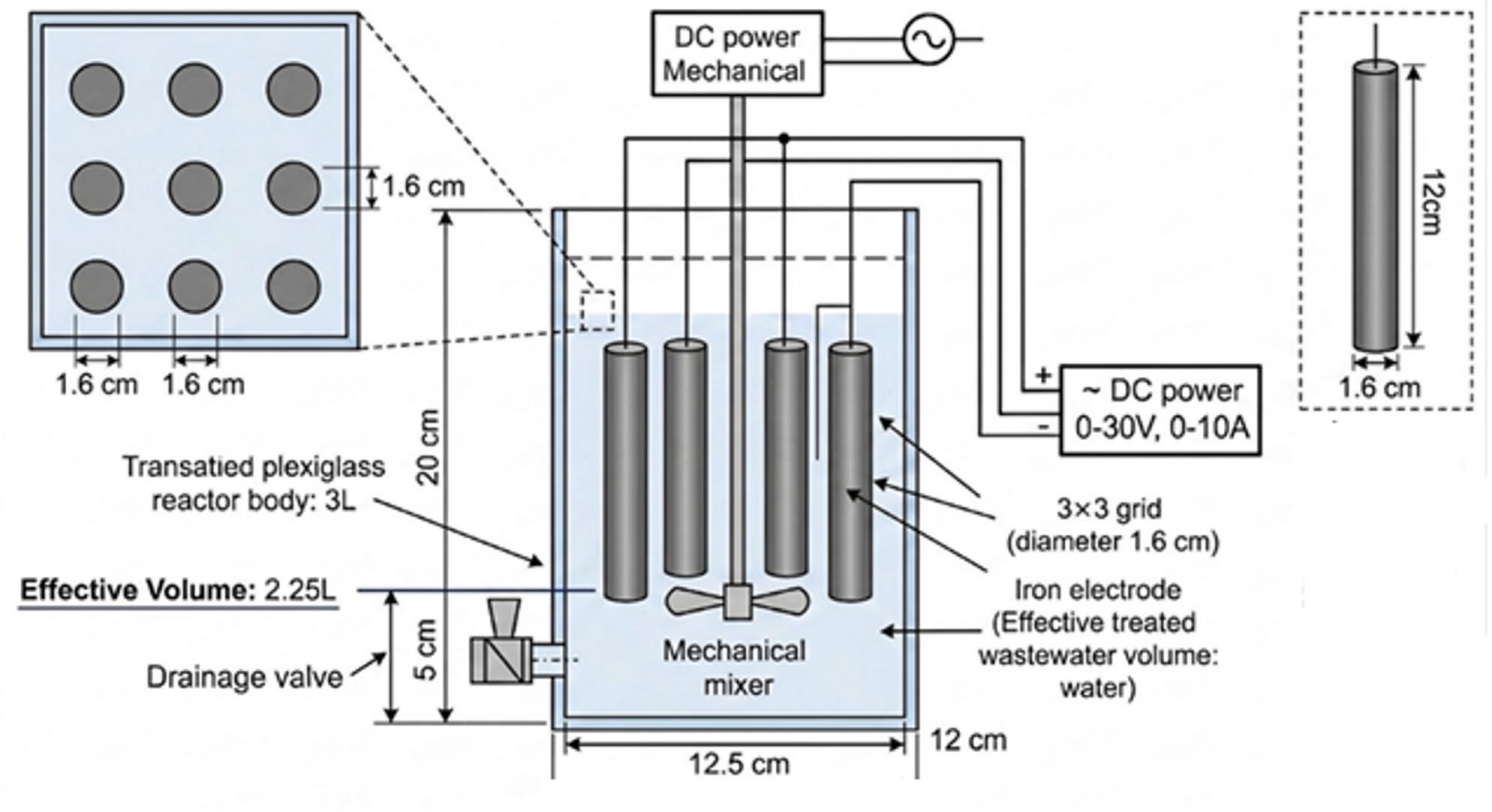
Electrocoagulation treatment cell.

A schematic diagram of the EC unit is shown in Fig. 1. The reactor body is a translucent plexiglass container with a volume of 3L, measuring 12.5 cm in length, 12 cm in width, and 20 cm in height. The drainage valve is positioned 5 cm above, yielding an effective volume of 2.25 L. Nine cylindrical iron electrodes were arranged in three rows, each containing three electrodes spaced 1 cm. When the distance between the electrodes increases, the resistance between them will also increase, requiring a greater effort to overcome it, and electricity consumption will also rise. Therefore, the distance between the electrodes should be appropriate, neither too large to increase the resistance nor too small, which would necessitate frequent polarity changes due to the repeated deposition of the resulting mass^14,25^. Numerous studies have advocated for a 1 cm distance between electrodes especially at medium electrical conductivity, as it promotes the development of coagulation materials, diminishes electrical consumption, and is deemed practicable^26–28^. Each electrode possesses a diameter of 1.60 cm and an effective height in water of 12 cm, yielding effective surface area 60.28 cm^2^ per electrode and a total volume of 24.11 cm^3^ per electrode. The cumulative volume of all electrodes is 0.217 L, while the effective volume of the treated wastewater in the electrochemical unit is around 2L. A mechanical mixer was positioned centrally within the apparatus, driven by a controlled DC power source. The DC power supply provides an output voltage range of (0–30 V) and a current range of (0–10 A). Experiments were performed on aluminum and iron electrodes to assess their efficacy in eliminating specified contaminants, consequently identifying the optimal electrode for purifying water from automotive manufacturing facilities.

### Operating conditions

The system was first operated under batch flow conditions in order to determine the optimum operating and design parameters. The first set of experiments was conducted to determine the required electrocoagulation contact time for iron electrode material. The reactor was operated at an initial pH 7 and a CD of 2 mA cm⁻². Samples were collected for analysis after 10, 20, 30, 40, 50, and 60 min of electrolysis for determination of Ni^+2^, Cr, and COD removal efficiencies. After determining the appropriate electrolysis time, the influence of initial pH on treatment efficiency was investigated. Experiments were carried out at pH values of 4, 7, and 9 while maintaining a CD of 2 mA cm^-2^ and the previously determined optimal electrolysis time. Removal efficiencies of Ni^+2^, Cr, and COD were evaluated to identify the most suitable pH for iron electrode material.

Subsequently, the effect of CD was examined. CD of 2, 3, 4, and 6 mA cm^-2^ were applied while maintaining the previously determined optimal pH and reaction time. Based on these experiments. Electrical energy consumption and electrode dissolution were calculated at the optimum conditions, and the variation of pH during the electrocoagulation process was monitored. Each experiment is conducted three times, and the average is taken from them, and a standard deviation of the results is also calculated. The sludge produced during EC at optimal operating conditions was collected and analyzed (SEM, EDX, XRD and FTIR analysis) to determine its composition and physio-chemical characteristics. Also generated sludge was quantified for sludge volume(SV), total solids (TS), and volatile solids (VS).

Following the optimization stage, the reactor was operated using continuous-flow and under optimal batch operating conditions. The purpose of this stage was to compare the treatment performance under continuous and batch operation modes. In addition to Ni^+2^, Cr, and COD, other water quality parameters were analyzed before and after treatment in case of continuous-flow conditions, including TSS, oil and grease, TP, TKN and BOD, to evaluate the effectiveness of the process in removing additional contaminants present in the wastewater. Additionally, the SV, TS and VS were quantified. The characterizations were conducted in accordance with the established protocols for the analysis of water and wastewater^29^.

### Calculations

The effectiveness of pollutant removal is calculated as a percentage based on the beginning and final concentrations of contaminants in wastewater. The operational cost (OC) is determined by the formula: OC = electricity consumption (EC) + electrode, where both electricity and electrode consumption refer to the amounts used per m^3^ of treated wastewater. Energy consumption is ascertained in accordance with Eq. (1):

1 $$Ec{\text{ }}\left( {KWh{\text{ }}m^{{ - 3}} } \right)\frac{{V*I*t}}{{v*1000}}$$

Where V represents the applied voltage, I denotes the current in amperes, t indicates the treatment duration in hours, and v signifies the amount of treated wastewater in m^3^.

Theoretical electrode consumption was estimated, whereas real electrode material consumption was determined in accordance with Faraday’s law as presented in Eq. (2):

2 $$Electrode{\text{ }}consumption{\text{ }}\left( {kg{\text{ }}m^{{ - 3}} } \right)\frac{{I*t*Mw}}{{z*F*~v*1000}}$$

Where F is Faraday’s constant, quantified as 96,485 C mol^− 1^; Mw is the molar mass of iron, measured as 55.845 g mol^− 1^; and z signifies the number of electron exchanges (z = 2). The actual electrode consumption was determined by measuring the mass differential of the electrodes before and after treatment over a certain volume within a set timeframe.

## Results and discussion

### Effect of contact time on the EC efficiency

**Fig. 2:**
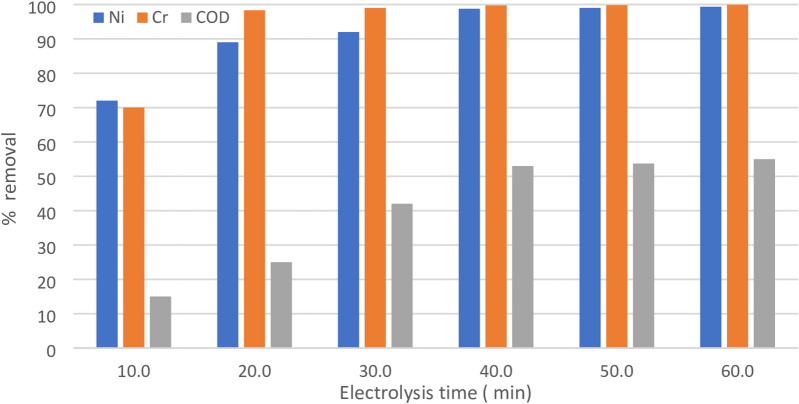
Effect of contact time on the removal efficiency.

The operational kinetics and purification performance of EC process are fundamentally governed by the electrolysis time, which directly dictates the stoichiometric theoretical dosage of coagulant species liberated from the sacrificial iron anodes according to Faraday’s law, as emphasized in the foundational frameworks of Verma et al.^17^, Al-Shannag et al.^30^, and Kabdaşlı et al.^31^. In this investigation, the influence of reaction time was systematically ±valuated under a constant applied CD of 2 mA cm^-2^ and an initial wastewater pH of 7 ± 0.20. As illustrated in Fig. 2, the removal efficiencies of Ni^+2^, Cr(VI), and COD exhibited monotonic increments as a function of prolonged electrolysis time. This observed trend is strictly ascribed to the continuous electrochemical generation of ferrous ions Fe^+2^ at the anode and hydroxyl ions OH^-^ at the cathode, fostering an environment conducive to massive flocculation, adsorption, and co-precipitation, which matches the mechanisms detailed by Verma et al.^17^, and Kurt et al.^32^.

**Fig. 3:**
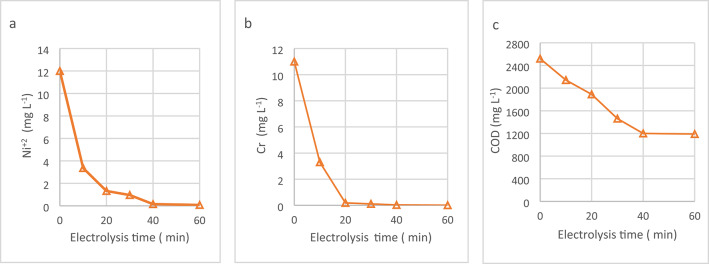
Effect of retention time on the reduction of a: Ni, b: Cr and c : COD.

The elimination of Ni^+2^ proceeded with rapid kinetics during the initial stages of the electrochemical reaction. As quantified in Fig. 3a, the residual concentration of Ni^+2^ dropped drastically from an initial baseline of 12 ± 0.4 to 3.8 ± 0.25 mg L^-1^ after 10 min of treatment, and further declined to 1.2 ± 0.11 mg L^-1^ by the 20 min, demonstrating compliance behavior similar to that reported by Akbal and Camcı^33^. Upon extending the electrolysis time to 40 and 60 min, the residual Ni^+2^ concentrations were suppressed to 0.15 ± 0.03 and 0.08 ± 0.01mg L^-1^ respectively, which comfortably meets the requirements of global clean water benchmarks.

Mechanistically, the immobilization of Ni^+2^ involves a synergistic combination of chemical precipitation and physical adsorption/enmeshment onto the in situ generated amorphous iron hydroxide flocs, a pathway thoroughly corroborated by Al-Shannag et al.^30^ and the comprehensive review of Bazrafshan et al.^34^. Concomitant with the progress of electrolysis, the bulk solution pH escalated to 8.8 ± 0.2 mg L^-1^ at 30 min and reached a stable plateau of 9.3 ± 0.15 mg L^-1^ at 40 min. This alkaline migration is driven by the accumulation of OH^-^ ions, which directly catalyzes the thermodynamic transition of soluble Ni^+2^ ions into highly insoluble nickel hydroxide complexes Ni(OH)_2_ within their optimal pH stability range^30–35^.

Simultaneously, the elimination of Cr(VI) exhibited the most rapid reduction and separation kinetics among all analyzed pollutants. As delineated in Figs. 2 and 3-b, the residual Cr(VI) concentration plummeted from an initial value of 11 ± 0.35 to 3.50 ± 0.18 mg/I within the first 10 min. At an electrolysis time of 20 min, the residual concentration reached 0.18 ± 0.04 mg L-1, which satisfies the stringent regulatory discharge ceiling of 0.50 mg L-1. By terminating the reaction at 40 min, total removal efficiency and residual Cr of 99.90 ± 0.05% and 0.011 ± 0.001 mg L^-1^ was attained respectively. This superior performance is elucidated by a well-documented sequential reductive-precipitation pathway identified by Verma et al.^17^ and Al Aji^36^. The electrochemically dissolved Fe^+2^ ions serve as a highly active reducing agent, driving the immediate homogenous reduction of toxic, highly mobile Cr(VI) into the less toxic trivalent state Cr(III)^17^. Subsequently, the newly formed Cr(III) cations interact with the abundant electro-generated OH^-^ ions to synthesize highly insoluble chromium hydroxide Cr(OH)_3_ and/or mixed (Fe-Cr) complex precipitates (Cr_x_- Fe_1-x_ OH_3_), as thoroughly validated by Verma et al.^17^ and Al Aji^36^. This empirical outcome aligns seamlessly with the findings of Al-Shannag et al.^30^, confirming that a retention time of 40 min is highly sufficient to achieve separation efficiencies exceeding 98% for multi-metal matrices containing Ni^+2^ and Cr ions.

In stark contrast to the heavy metal ions, the reduction of COD followed a significantly slower degradation trajectory, as depicted in Fig. 3-c. From an initial heavy organic loading of 2500 ± 75 mg L^-1^, the residual COD reached 2150 ± 62 and 1460 ± 45 mg L^-1^ after 10 and 30 min of electrolysis. The structural elimination of COD during iron-based EC is primarily governed by charge neutralization of colloidal organic fractions and subsequent physical entrapment within the growing polymeric iron hydroxide matrix, as defined by Al-Shannag et al.^30^ and Bellebia et al. ^37^. The electro-generated iron flocs required a prolonged operational duration of 40 min to develop sufficient spatial density, surface area, and active adsorption sites to maximize organic capture, eventually lowering the residual COD concentration to 1200 ± 48 mg L^-1^. Similar slow organic extraction dynamics in electrocoagulation treatment have been extensively reviewed by Kabdaşlı et al.^31^, Bellebia et al.^37^ and Yavuz and Ögütveren^38^.

Importantly, prolonging the electrolysis time beyond 40 min did not yield any statistically significant improvement in the organic removal efficiency. Despite this notable reduction, the final residual COD concentration remained above the regulatory direct-discharge standard of 1100 mg L^-1^, a behavior that mirrors the challenges noted by Ni’am et al.^39^ and Kurt et al.^32^ regarding heavily loaded organic streams. Consequently, optimizing higher current densities or implementing secondary oxidation/adsorption mechanisms in subsequent experimental phases is imperative to enhance the floc-adsorption capacity and guarantee absolute compliance with environmental legislation.

**Fig. 4:**
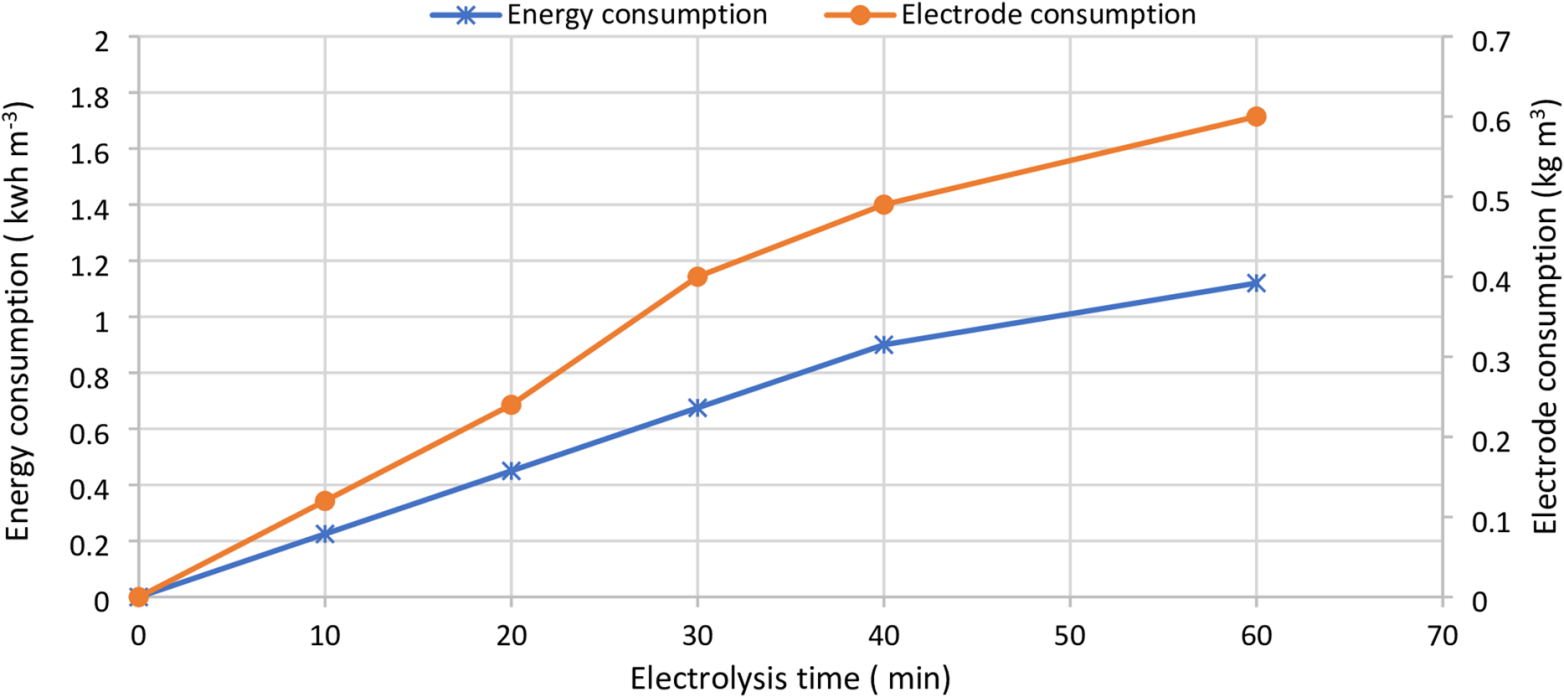
Relationship between energy and electrode usage over time throughout the EC.

Based on the experimental chronological runs, the removal efficiency of COD exhibited a rapid initial degradation phase during the first 40 min of electrolysis, after which the degradation rate reached a plateau or experienced a minor decline up to 60 min which the residual COD reached to 1190 +25 mg L^-1^. This behavior aligns with the pseudo-first-order kinetic model frequently used to describe organic matter oxidation and co-precipitation in electrocoagulation systems. In the early stages of the process, the high availability of freshly generated active coagulant species, coupled with high initial organic concentrations, drives a rapid reaction rate. However, as the reaction time approaches 40 min, the bulk concentration of organic contaminants drops significantly, shifting the system toward its equilibrium state (Ce). At this juncture, the rate of COD trapping onto the flocs balances out, resulting in a kinetic plateau. This stagnation and the slight subsequent decline in efficiency can be further interpreted by examining the evolution of the solution’s pH, which rose from value of 9.3 ± 0.15 at 40 min to 9.80 ± 0.1 at 60 min. The continuous generation of (OH-) via water reduction at the cathode promotes a highly alkaline environment. Prolonging the treatment time to 60 min induces an over-treatment phenomenon. At a pH of 9.80 ± 0.1, the amphoteric nature of the electro generated metal hydroxides favors the formation of soluble monomeric and polymeric anions (such as Fe(OH)_4_^–^). These soluble species lack the dense polymeric structure required for the effective adsorption and sweeping of organic molecules contributing to COD. Consequently, the alkaline shift not only restricts further COD removal but can also lead to the partial dissolution of pre-formed flocs, re-releasing some trapped organic fragments into the solution as emphasized in the foundational frameworks of Al-Shannag et al.^30^. And the reason could be the formation of complex organic materials, which requires increasing the initial current intensity before raising the pH above 9, as also explained by Al-Shannag et al.^30^. Using another type of treatment after electrocoagulation is necessary in case the increase in CD is insufficient to enhance the required efficiency or to minimize the remaining COD, such as using strong anodic oxidation, which is effective in complex organic materials, as the scientist mentioned by Linares-Hernández et al.^40^. Thus, operation beyond 40 min provides no kinetic advantage and significantly escalates energy and electrode consumption costs. From an economic and operational perspective, Fig. 4 illustrates the cost-benefit optimization profile of the process. At the established 40 min threshold, the electrical energy consumption was recorded at 0.90 ± 0.05 kWh m^-3^, while the specific sacrificial electrode mass loss reached 0.49 ± 0.03 kg m^-3^. Further operational extensions of the electrolysis time were deemed economically non-viable, as they exponentially exacerbated operating costs through excessive power consumption and rapid electrode degradation without offering any meaningful gains in pollutant removal, which is similar, according to the discussion set by Al-Shannag et al.^30^ and Yavuz and Ögütveren^38^. Consequently, an electrolysis time of 40 min was selected as the optimal benchmark and maintained as a constant parameter for the subsequent experimental sequences dedicated to optimizing the initial pH and applied CD.

### Influence of initial pH on EC efficiency

**Fig. 5:**
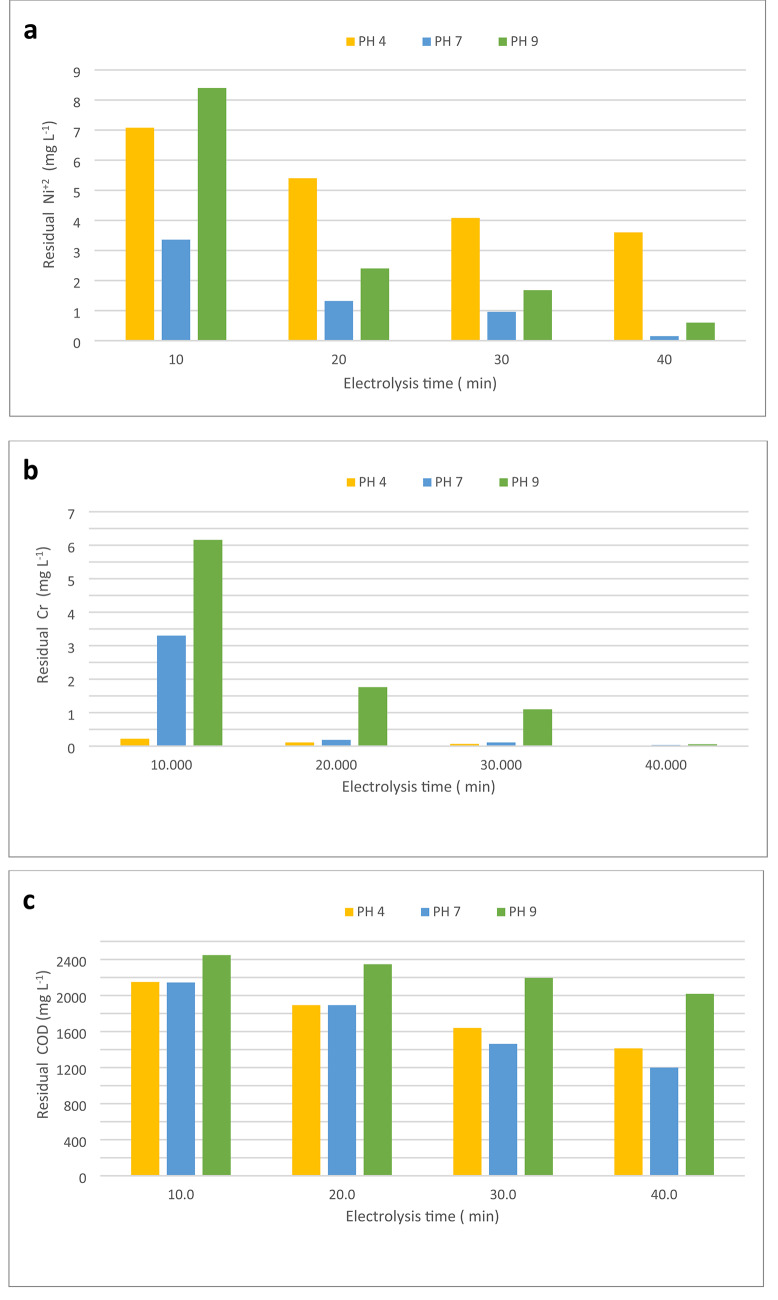
Impact of pH on the reduction of: (a) Ni, (b) Cr, and (c) COD.

After establishing the perfect reaction length of 40 min in the preceding experimental phase, the present part of this investigation aims to ascertain the ideal beginning pH for the (EC) process. The initial pH is a crucial regulating factor as it determines the speciation of metal ions and the structural development of the resultant coagulant flocs. Experiments were performed at a constant CD of 2 mA cm^-2^ within an initial pH range of 4.0, 7.0, and 9.0. As shown in Fig. 5-a, the system achieved a maximum Ni^+2^ removal efficiency of 98.74 ± 0.15% at an initial pH of 7.0 as mention previously. At a pH of 4.0 ± 0.20, Ni^+2^ demonstrated its minimal removal efficiency, which is equal 70 ± 1.2% due to its high solubility as Ni^+2^ ions. In these low pH settings, iron species predominantly exist as soluble ionic forms, which do not possess sufficient solid surface area for trapping. Ni^+2^ removal. At alkaline pH of 9.0 the efficiency was high which equal 95 ± 0.7% due to the natural precipitation of Ni (OH)_2_, but the neutral pH of 7.0 demonstrated greater efficacy. This phenomenon is ascribed to the interplay between chemical precipitation and physical adsorption onto the iron matrices^41^.

In Fig. 5-b, the system demonstrated optimal performance for Cr(VI) removal at an initial pH of 7.0 ± 0.20, achieving a concentration reduction of 0.03 ± 0.01 mg L^-1^ as mention previously. Mollah et al.^42^ determined that at neutral pH, the electrolytic dissolution of the iron anode generates a complex combination of Fe(OH)_2_ and Fe(OH)_3_, which fosters the production of “Green Rust,” hence accelerating the reduction of Cr(VI) to Cr(III). At a pH of 4.0 ± 0.20, Chromium removal was substantial, since the acidic conditions facilitate the conversion of Cr(VI) to Cr(III). This process is a proton-consuming redox reaction; thus, the elevated concentration of H^+^ ions at pH 4.0 serves as a catalyst, expediting the transformation of soluble chromate into Cr(III) ions, which subsequently precipitate when the pH naturally rises during electrolysis^17^. The removal kinetics at pH of 9.0 ± 0.20 were significantly inhibited, but after 40 min, the removal was 99.50%. This slowdown is primarily attributed to the dominance of soluble chromate species (CrO_4_^-2^) and the formation of more stable and less reactive anionic iron complexes such as Fe(OH)_4_^-^. Moreover, the increase in the concentration of OH^-^ ions reduces the availability of adsorption sites on the surface of iron hydroxide blocks^8,17^.

The influence of pH on COD reduction exhibited a similar pattern (Fig. 5-c), with a peak reduction at pH 7.0 ± 0.20 as mention previously, in contrast to 44 ± 2% at pH 4.0 ±  0.20 and merely 20 ± 3.1% at pH 9.0 ± 0.20. According to Al-Shannag et al.^30^, the reduced efficiency at pH higher 9.0 ± 0.20 is likely attributable to the diminished stability of the coagulant flocs and the competing adsorption of organic molecules and OH^-^ ions for active sites. Observing the pH during electrolysis demonstrated a self-buffering effect; the pH consistently increased in all experiments due to the generation of OH^-^ at the cathode. The trial initiated at pH 7.0 ± 0.1 attained a final value of 9.6 ± 0.15, a range that guarantees the ultimate stability and precipitation of remaining contaminants into the sludge phase. The results indicate that a pH of 7.0 is the ideal beginning setting for the iron-based EC procedure. This value guarantees optimal elimination of heavy metals and organic contaminants while circumventing solubility complications associated with acidic conditions and ionic interference common in alkaline settings. Osipenko and Pogorelyi, Zongo et al.^43^, and Bazrafshan et al.^44^, have demonstrated that the optimal operating pH for maximizing the performance of iron-based electrocoagulation systems lies within the neutral to slightly alkaline range of 6.0–9.0. Thus, optimal pH of 7.0 will be employed in the next part of this study to examine the ideal CD, with the objective of maximizing COD removal and ensuring the final effluent quality complies with regulatory standards for industrial discharge.

### Impact of applied CD

**Fig. 6:**
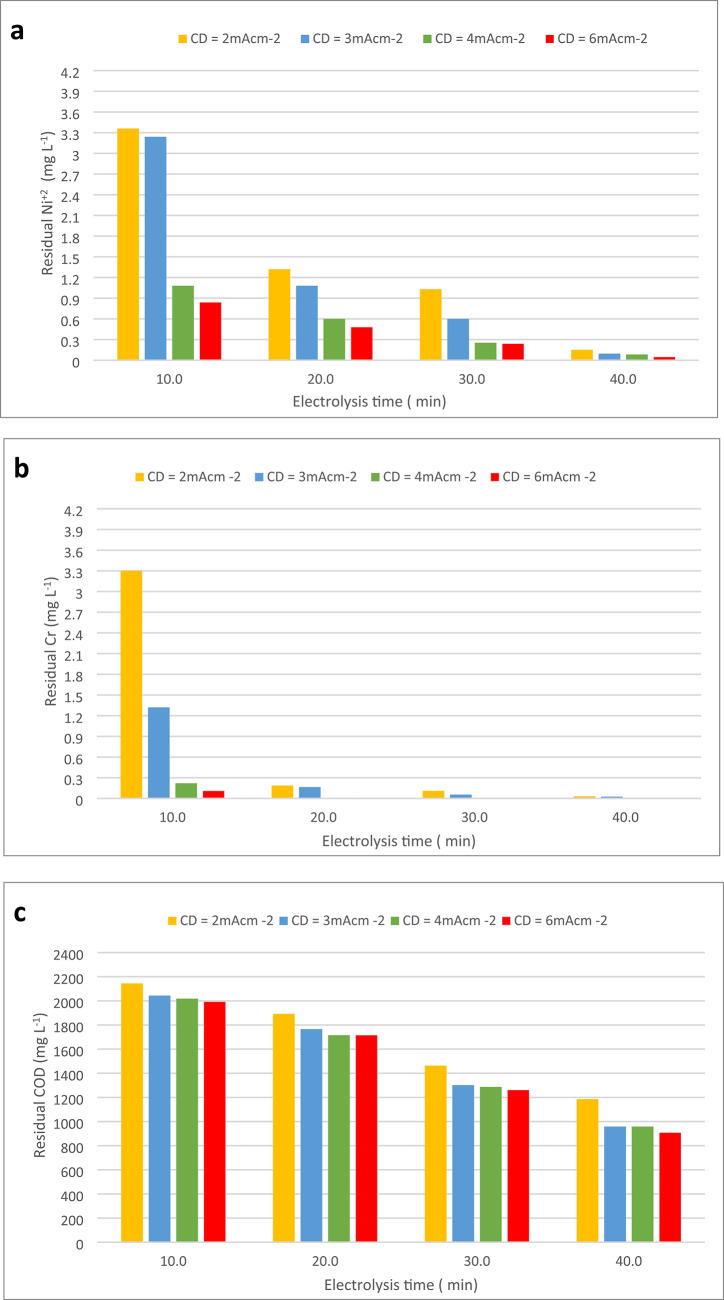
Impact of CD on the reduction of: (a) Ni, (b) Cr, and (c) COD.

After establishing the appropriate reaction length of 40 min and an initial pH of 7.0 in previous experiments, the CD phase of this investigation aims to determine the ideal CD to be applied. CD is a critical operational parameter in EC, since it directly influences the rate of coagulant dose, bubble generation, and the overall kinetics of pollutant elimination. This study examined the impact of different current densities (2, 3, 4, and 6 mA cm^− 2^) to guarantee concurrent compliance for all targeted pollutants. Concerning Ni^+2^ removal (Fig. 6-a), the system exhibited significant effectiveness at the lowest tested CD of 2 mA cm^− 2^ as mention previously, resulting in residual amounts of 1.02 ± 0.04 and 0.15 ± 0.0.03 mg L^− 1^ after 30 and 40 min. As the CD rose to 3 mA cm^− 2^, the removal efficiency remained elevated with residual amounts of 1.08 ± 0.05, 0.60 ± 0.07 and 0.09 ± 01 mg L^− 1^ after 20, 30 and 40 min respectively, whereas subsequent increases to 4 and 6 mA cm^− 2^ yielded minimal enhancements, suggesting that the requisite pH threshold for Ni^+2^ precipitation had already been attained. Increasing the CD to 3 mA cm^− 2^ reduces the electrolysis period to 30 min, successfully lowering Ni^+2^ residues to meet the environmental limit of 1.0 mg L^− 1^, compared to 40 min.

**Fig. 7:**
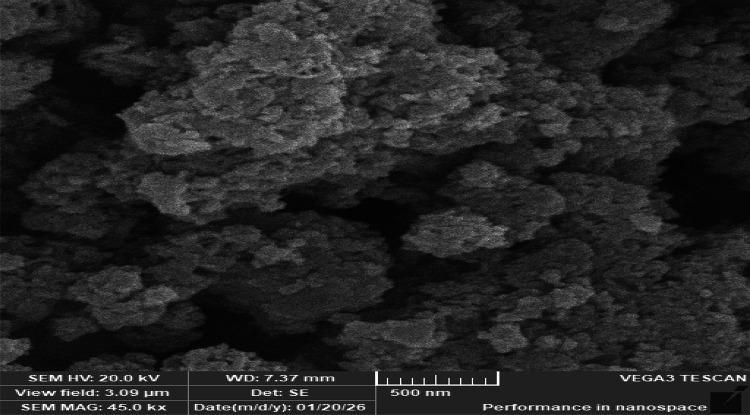
SEM for Sludge collection.

A comparable trend for Cr(VI) elimination noted (Fig. 6-b). At a CD of 2 mA cm^− 2^, the residual concentration decreased to 0.187 ± 0.02, 0.11 ± 0.02 and 0.03 ± 0.1 mg L^− 1^ as mention previously for 20,30 and 40 min respectively. The increase to 3 mA cm^− 2^ facilitated a more substantial production of coagulant species but not reduction the suitable electrolysis time smaller than 20 min. The residual concentration decreased to 1.32 ± 0.02, 0.615 ± 0.02, 0.05 ± 0.1 and 0.025 ± 0.01 mg L^− 1^ at 10, 20,30 and 40min respectively. Excessive current densities of 4 and 6 mA cm^− 2^ resulted residual concentration 0.22 ± 0.02 and 0.11 ± 0.01 mg L^− 1^ respectively at electrolysis time 10min. The applied CD was the critical factor for regulatory compliance in COD elimination (Fig. 7-c). At 2 mA cm^− 2^, the residual COD was 1200 ± 45 mg L^− 1^ as mention previously at 40min, which did not comply with the mandated discharge limit. Increasing the CD to 3 mA cm^− 2^ resulted in a greater flux of iron ions and achieved a pH of 9.80 ± 0.1 at 40min, so enhancing the adsorption capacity of the flocs and effectively reducing the COD to 940 ± 15 mg L^− 1^ at 40 min. Increasing the CD to 3 mA cm^− 2^ do not reduction the suitable electrolysis time than 40min, as residual COD after 30min reached to 1301 ± 35 mg L^− 1^. Subsequent increases to 4 and 6 mA cm^− 2^ did not result in significant enhancements in COD reduction. We conclude that the removal of COD is the decisive factor in determining the required CD and electrolysis time due to its complex organic composition compared to heavy metals. The deduced CD and electrolysis time are very suitable compared to previous research. “Demirci, Pekel, and Alpbaz.^45^, demonstrated a 64% removal efficiency during the electrocoagulation of authentic textile dyeing wastewater. This was achieved utilizing iron electrodes over a 60 min electrolysis time at a fixed CD of 10.4 mA cm^− 2^.

“Tezcan Un and Aytac.^46^, investigated the electrocoagulation of real textile wastewater using a packed bed electrochemical reactor with iron electrodes at current densities ranging from 20 to 50 mA cm^− 2^, achieving a maximum COD removal efficiency of 96.88% within electrolysis time of 90 min. In conclusion, following the concurrent assessment of all contaminants, a CD of 3 mA cm^− 2^ is designated as the best parameter. Although 2 mA cm^− 2^ sufficed for the abatement of Ni^+2^ and Cr, a current of 3 mA cm^− 2^ was requisite to attain the required COD decrease. The ideal criterion, along with an initial pH of 7.0 and a reaction duration of 40 min, ensures the highest efficiency in treating this particular industrial effluent. To minimize the residual COD to the lowest feasible concentration, a utilizing integrated approaches must be used like electro-oxidation(EO) with active chlorine, electro-Fenton (EF), and innovative light-assisted techniques such as photo electrochemical (PEC), solar photo electrochemical (SPEC), pulsed electric field (PEF), and solar pulsed electric field (SPEF), suggesting that their implementation in industrial waste^47^. For example, Meena et al.^48^ evaluated a hybrid electrocoagulation-electro oxidation (EC-EO) process utilizing a Ti/RuO_2_-IrO_2_ anode to treat synthetic tannery wastewater. Before full hybrid optimization, the standalone EC process was insufficient, achieving COD removal efficiency of only 69.78% under optimal conditions of (0.80 A, pH 6.9, and 65 min). After optimization of the hybrid system using a Central Composite Design (CCD), the COD removal efficiency significantly improved to 79.04% at a reduced current of 0.702 A and an extended treatment time of 110 min.

### Characterization of sludge produced at optimum operation

Starting with the quantitative production metrics, (SV) was 123 ± 12 L m^-3^, (TS) and (VS) content was of 1.57 ± 0.18 and 0.819 ± 0.10 kg m^-3^ respectively, that indicate a robust precipitation of iron-based coagulants. This values consider suitable and small according to sludge quantity produced by conventional treatment^49^. The (VS) content of approximately 51% is particularly significant; it confirms that over half of the sludge mass consists of organic matter and volatile complexes removed from wastewater. Ratio (VS/TS) around 0.50 is indicative of high organic loading and effective adsorptive micellar flocculation, distinguishing this EC sludge from purely inorganic chemical sludge and highlighting the successful removal of organic pollutants (COD).

Regarding the SEM micrograph, the morphology reveals a highly porous, uneven, and aggregated floc structure (Fig. 7). This “cauliflower-like” arrangement is characteristic of iron hydroxides formed during the rapid oxidation of sacrificial iron electrodes. As supported by the observations of Aragaw, T. A., & Aragaw, B. A.^50^, such extensive porosity provides a massive specific surface area, which is essential for the physical entrapment of organic macromolecules and suspended solids.

**Fig. 8:**
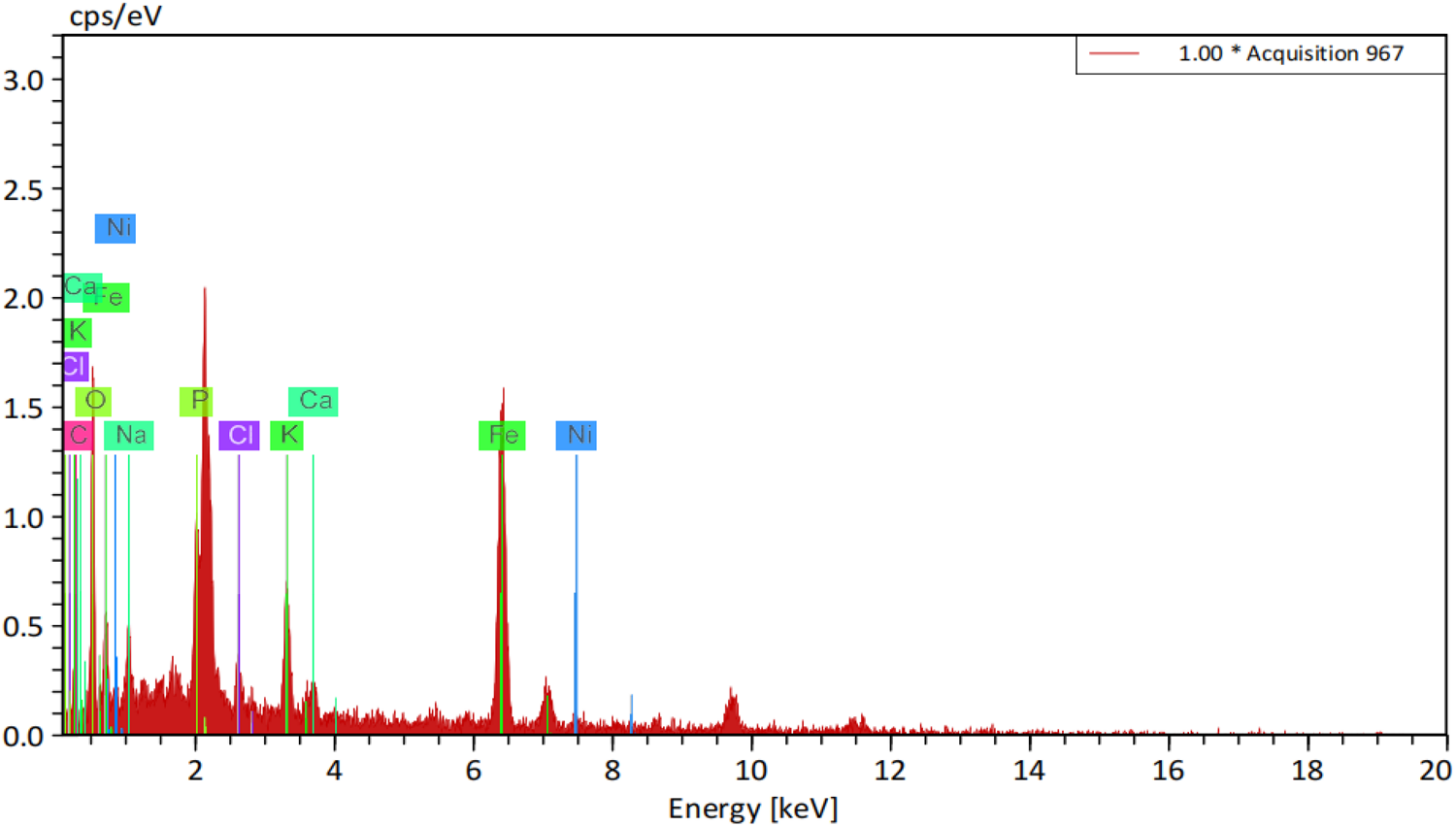
EDX for sludge collection.

This structural evidence is further validated by the EDX spectrum (Fig. 8); the dominance of Iron (43.36%) and Oxygen (27.71%) proves the formation of a resilient Fe(OH)n or FeOOH matrix. Furthermore, the detection of Ni^+2^ (1.68%) and Phosphorus (P) (6.52%) in the EDX data serves as empirical proof of “solid-phase transfer” where dissolved pollutants are successfully integrated into the sludge matrix via chemical precipitation and co-precipitation, consistent with the elemental analysis of EC by-products reported by Elazzouzi et al.^51^.

**Fig. 9:**
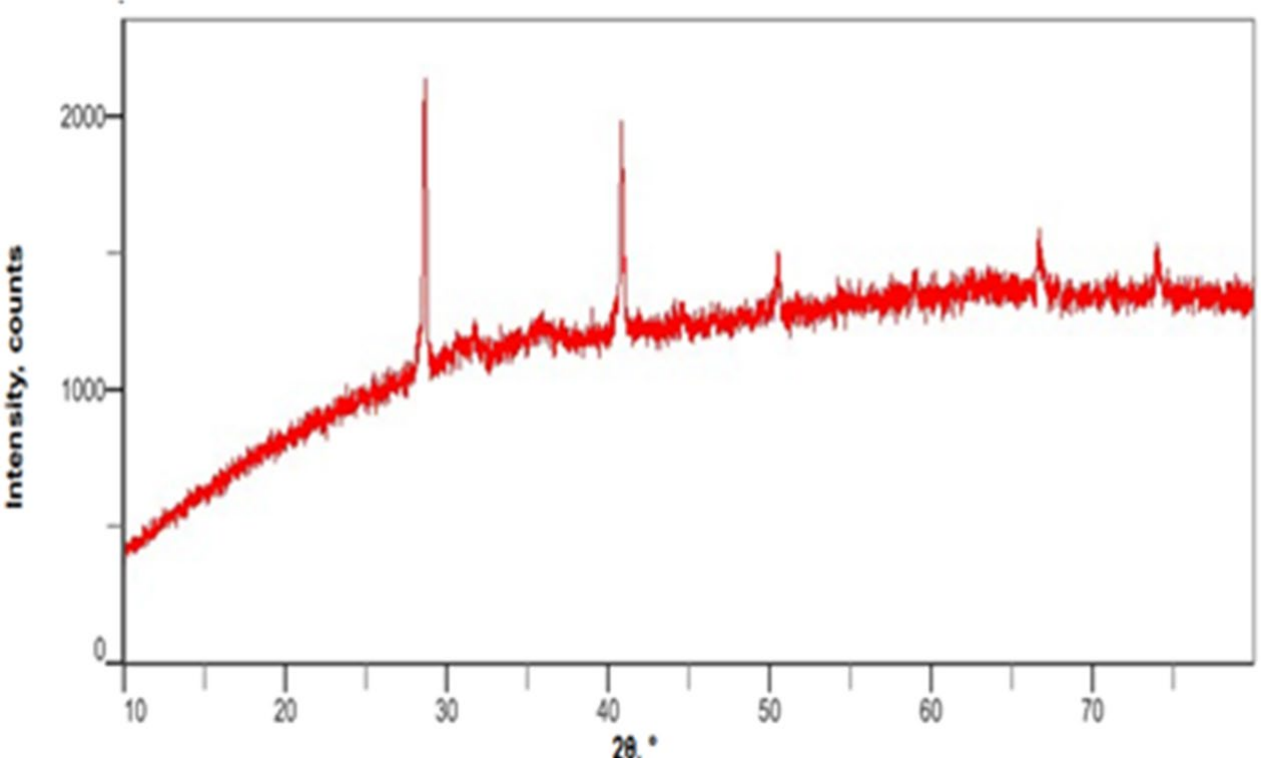
XRD for sludge collection.

The X-ray diffraction (XRD) pattern of the sample demonstrates a series of well-defined diffraction peaks, confirming a high degree of crystallinity (Fig. 9). Qualitative phase identification reveals a complex multicomponent material primarily composed of iron and chromium oxides, which are characteristic of the precipitates formed during the electrocoagulation process. The analysis indicates that iron-based phases have replaced aluminum as the primary structural matrix. The dominant crystalline phases identified include Magnetite (Fe_3_O_4_), and Hematite (Fe_2_O_3_), alongside significant chromium-bearing phases such as Eskolaite (Cr_2_O_3_) and Chromium Dioxide (CrO_2_). Additionally, Ni^+2^-related phases, specifically Nickel Oxide (NiO) and Nickel Dioxide (NiO₂), were detected, contributing to the structural complexity of the material. The presence of diffraction peaks corresponding to Magnetite (Fe_3_O_4_) and Hematite (Fe_2_O_3_) suggests the transformation of primary hydroxides into more stable crystalline oxides. According to Aragaw, T. A., & Aragaw, B. A.^50^. The most prominent diffraction peak is observed at an angle of 34.53° (2θ) with a normalized intensity of 100.00, which is principally indexed to (Fe_0.945_ O) and (NiO) phases. Another significant peak appears at 28.626° (2θ) with a normalized intensity of 32.25, attributed to (CrO_2_). The presence of (Fe_3_O_4_) is further confirmed by a distinct peak at 66.64° (2θ).

From a crystallographic perspective, (Fe_3_O_4_) belongs to the cubic crystal system with lattice parameters a = b = c = 8.4045 Å (α = β = γ = 90.00°), where the primary diffraction occurs at the (311) plane. .(Fe_2_O_3_) crystallizes in a rhombohedral system with lattice parameters a = b = c = 5.5398 Å and angles of 55.23°, with its most intense peak associated with the (211) plane at 32.47° (2θ). Regarding the chromium phases, (Cr_2_O_3_) exhibits a hexagonal crystal system (a = b = 4.9128 Å, c = 13.4524 Å), with its characteristic peak at 33.95° (2θ) corresponding to the (104) plane. (CrO_2_) follows a tetragonal arrangement, showing a primary peak at 28.58° (2θ). The coexistence of these diverse metallic oxides reflects the intricate electrochemical oxidation and co-precipitation mechanisms inherent in the treatment process, where the sacrificial iron anode interacts with metallic pollutants to form stable mineralized phases. This amorphous nature is highly beneficial for the sludge’s potential as an adsorbent; as demonstrated by Rajaniemi et al.^52^, disordered phases typically possess more active surface sites for pollutant binding than fully crystalline structures.

**Fig. 10:**
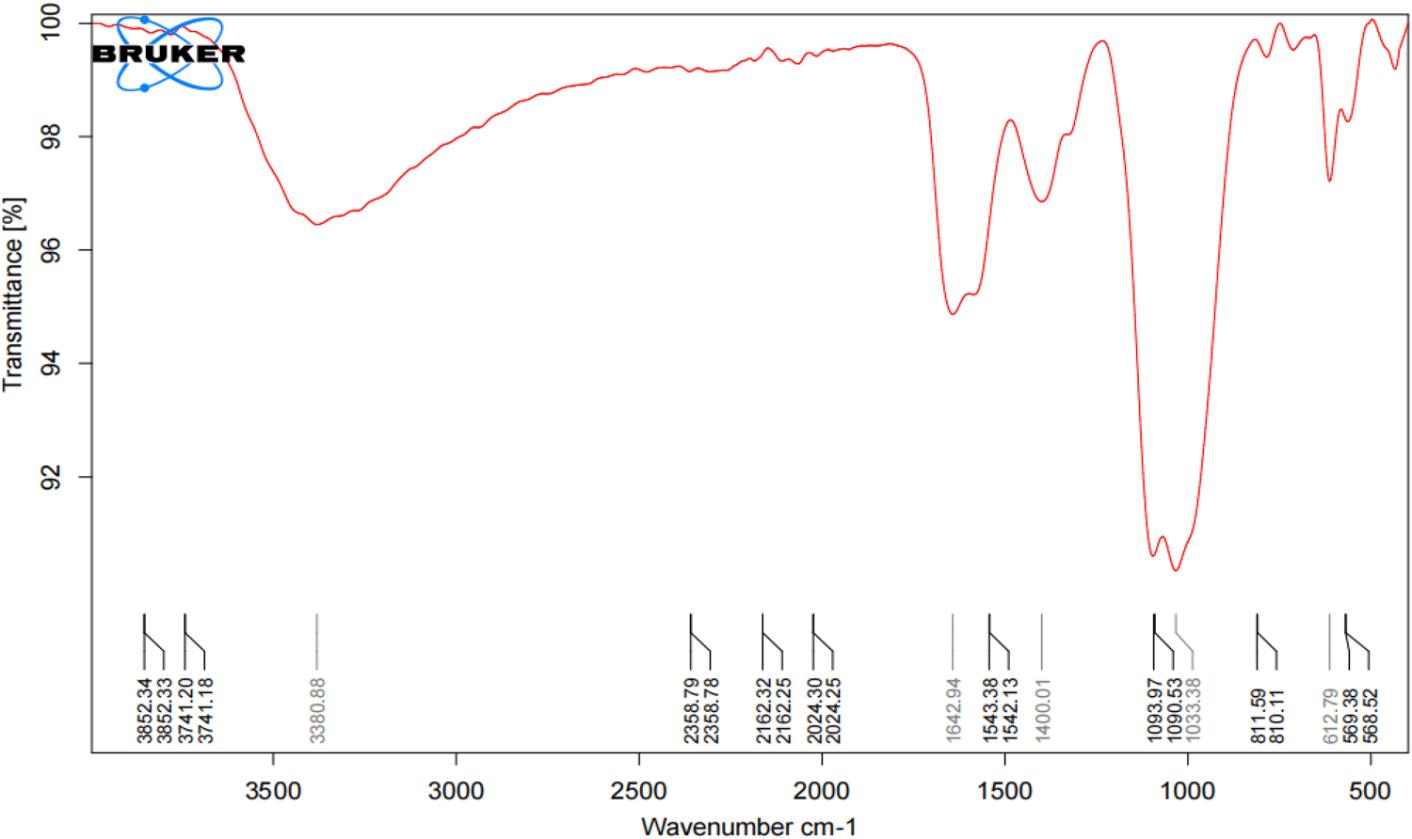
FTIR for sludge collection.

Complementing these findings, the FTIR spectrum identifies the functional groups responsible for chemical binding (Fig. 10). The broad absorption band at 3380 cm^–1^ represents the stretching vibrations of hydroxyl groups (-OH), which are essential for surface complexation and ion exchange. The distinct peaks at 612 cm^–1^ and 568 cm^–1^ are assigned to Fe-O lattice vibrations, confirming the structural integrity of the iron oxide backbone, which is consistent with the FTIR characterizations performed by Aragaw, T. A., & Aragaw, B. A.^50^. Collectively, these analytical results prove that the electrocoagulation process effectively mitigates pollutants through a synergy of charge neutralization, chemical precipitation, and surface adsorption within a stable iron-rich matrix.

Based on the aforementioned findings, it can be concluded that the generated sludge exhibits a hybrid semi-crystalline nature, characterized by well-defined crystalline oxide phases (e.g., Fe_3_O_4_and Fe_2_O_3_) synthesized on a highly disordered, amorphous iron hydroxide backbone. This coexistence is mechanistically advantageous; the crystalline domains confirm the definitive phase transformation of the mineralized pollutants, while the structurally unrefined, amorphous fractions provide an abundance of active surface coordinates, optimizing the thermodynamic capacity for adsorptive physical entrapment.

### Techno-economic assessment of the EC process

Following the identification of the optimal operating conditions from batch experiments, a continuous flow system was implemented to evaluate the process’s efficiency on a larger scale and ensure its reliability for industrial applications. The continuous system was operated using a pump flow rate of 45 to 50 mL min^-1^. Under optimal batch operating conditions (initial pH of 7.0 ± 0.20, a CD of 3 mA cm^-2^ and electrolysis time of 40–45 min, the continuous flow system demonstrated high performance in removing a broad spectrum of pollutants. As summarized in Table 2, which details the wastewater characteristics before and after treatment, the results showed that COD was successfully reduced from 2500 ± 75 to 915 ± 15 mg L^-1^. Additionally, Cr and Ni^+2^ reached final concentrations of 0.15 ± 0.01 and 0.2 ± 0.02 mg L^-1^ respectively, both falling well within the safe environmental discharge limits of 0.5 and 1 mg L^-1^ for Cr and Ni^+2^ respectively. The residues of other pollutants after treatment, including suspended solids, biochemical oxygen demand, phosphorus, and oils and greases, were below the permissible limit according to environmental law (details in Table 2). From an economic perspective, the techno-economic evaluation focused on resource consumption at the optimal CD of 3 mA cm^-2^, which served as the basis for the final cost calculation. At this setting, the energy consumption reached 1.65kWh m^-3^, while the electrode consumption was recorded at 0.60 kg m^-3^. Electricity consumption is deemed minimal in comparison to earlier research’s^34^ for example, Ebba et al.^53^ evaluated the treatment performance and operational costs of (EC) process using Fe–Fe and Al–Al electrode configurations. The major operating parameters including pH (3–7.50), current (0.03–0.09 A), electrolyte concentration (1–3 g L^-1^ and electrolysis time (20–60 min) significantly influenced pollutant removal efficiencies and energy requirements. Under the final optimized parameters using Fe–Fe electrodes, which was 0.09 A, electrolyte concentration of 3 g L^-1^ and pH of 7, the system maximized performance, achieving 94.40, 97.02 and 90.91% removal for color, COD and turbidity respectively. Economically, the optimized process required an energy consumption of 36 kWh m^-3^. The use of 3 mA cm^-2^ is technically justified as it is the necessary threshold to achieve the mandatory COD reduction below the 1,100 mg L^-1^ limit. Based on market prices (Electricity: 0.18 USD kWh^-1^ and Iron: 106 USD ton^-1^, the total operating cost for treating one m^-3^ of wastewater under these optimal conditions was calculated to be approximately 0.36 USD m^-3^ which is deemed minimal in comparison to earlier research’s^54^ for example, Kobya et al.^55^ treated high-strength metalworking wastewater using a continuous electrocoagulation process, proving that Iron (Fe) electrodes are more efficient and cheaper than Aluminum (Al). Under optimized conditions which was CD of 90 A m^-2^ and electrolysis time 70 min, the Fe-system maximized removals to 94.3% COD, 90.1% TOC, and 99.3% turbidity. Economically, Fe electrodes lowered baseline operating costs to 1.03–3.80 USD m^-3^ (compared to 1.37–4.74 USD m^-3^ for Al), while the total cost under final optimized parameters—including energy, electrode consumption, chemicals, and sludge disposal was minimized to 3.09 USD m^-3^ (2.63 USD/kg removed COD). Ozyonar and Karagozoglu^56^ treated slaughterhouse wastewater using a batch electrocoagulation reactor, comparing Al and Fe electrodes. Under optimized conditions (100 A m^-2^ for 20 min), the Al system (pH 4) achieved 78.3% COD removal, while the Fe system (pH 6) achieved 76.7% COD and a superior 95.9% turbidity removal. Economically, Fe electrodes were significantly cheaper, reducing the operating cost to just 0.872 USD m^-3^ compared to 2.757 USD m^-3^ for Al, while outperforming conventional chemical coagulation. This analysis confirms that the continuous flow electrocoagulation process, at the specified flow rate and CD, is not only technically robust in ensuring full environmental compliance but is also an economically viable solution for industrial-scale wastewater treatment. Also for hybrid system, the cost for m^3^ treatment and electricity consumption in our research is much lower. For example, the Meena et al.^57^ investigated a novel, continuous-mode hybrid system combining electrocoagulation (Fe/Fe electrodes) and electro oxidation (Ti/TiO2 -IrO2-RuO2 anode) to treat synthetic tannery wastewater. Optimizing the process via a Box-Behnken design, the integrated system achieved notable removal efficiencies: 75.97% for 4-chlorophenol, 82.33% for Cr(VI), 89.00% for COD, and 37.30% for TOC. Economically, the hybrid treatment demonstrated an energy consumption of 28.85 kWh m^-3^ with an estimated operating cost of 2.11 USD m^-3^, highlighting its potential as a scalable and innovative approach for industrial wastewater management.

**Table 2:** Wastewater characteristics before and after continuous EC treatment.

Parameters	Unit	Raw wastewater	EC treated wastewater	Discharge limits
TSS	mg L^− 1^	500 ± 150	121 ± 17	800
COD	mg L^− 1^	2500 ± 75	915 ± 15	1100
BOD	mg L^− 1^	1150 ± 150	430 ± 40	600
TKN	mg L^− 1^	17 ± 7	13 ± 1.4	100
Oil and grease	mg L^− 1^	160 ± 25	80 ± 6.5	100
TP	mg L^− 1^	25 ± 5	7 ± 2.2	25
Cr	mg L^− 1^	11 ± 0.30	0.15 ± 0.01	0.50
Ni^+2^	mg L^− 1^	12 ± + 0.40	0.2 ± 0.02	1

## Conclusion

The primary objective of this study was to evaluate the technical and economic viability of treating complex automotive manufacturing wastewater containing high organic loads and toxic heavy metals Ni^+2^, Cr(VI), and COD using electrocoagulation. The core novelty of this work lies in the development of a distinct, easy to implement and optimized iron electrode configuration represented by circular iron electrodes instead of square or rectangular plates. This innovative geometry was integrated with optimized dynamic parameters to maximize pollutant sequestration while minimizing resource consumption.

Batch experiments were conducted using iron electrodes on real effluent characterized by baseline concentrations of 12 ± 0.40, 11 ± 0.30 and 2500 ± 75 mg L for Ni^+2^, Cr(VI) and COD respectively. Through systematic optimization, the critical design criteria for a scalable system were established at an initial pH of 7, (CD) of 3 mA cm^-2^ and an electrolysis time of 40 min. These optimal parameters successfully balanced peak treatment efficiency with the lowest possible electrode consumption and electrical energy demand. The residual Ni^+2^, Cr, COD was 0.096 ± 0.02, 0.025 ± 0.01 and 940 ± 15 mg L^-1^ respectively. Transitioning from batch to a continuous-flow regime under optimal batch operating conditions yielded residual concentrations of Ni^+2^, Cr and COD was 0.20 ± 0.02, 0.15 ± 0.01 and 915 ± 15 mg L^-1^ respectively, achieving full compliance with stringent national environmental discharge regulations.

When connected to existing literature, the performance and economic metrics of this implemented system are highly acceptable. The geometrically optimized configuration successfully minimized electrical energy consumption to a mere 1.65 kWh m^-3^ and specific electrode depletion to 0.60 kg m^-3^. Consequently, the final operating cost was reduced to a highly competitive 0.36 USD m^-3^, which represents a highly viable and optimized expenditure for industrial-scale applications compared to conventional electrochemical treatments.

Furthermore, comprehensive microstructural and spectroscopic analysis (SEM/EDX, XRD, and FTIR) resolved the actual nature of the generated sludge, confirming a hybrid semi-crystalline structure where robust crystalline mineral frameworks (primarily Magnetite, Fe_3_O_4_, and Hematite, Fe_2_O_3_) are synthesized over a disordered, amorphous iron hydroxide matrix. This specific dual morphology offers an advantageous mechanistic synergy: the crystalline domains permanently immobilize the hazardous heavy metals via solid-phase transfer, while the coexisting amorphous fraction provides an abundance of active surface sites that maximize the adsorptive trapping of complex organic pollutants (COD). Ultimately, the derived design framework and novel electrode geometry deliver a technically robust, economically sustainable, and implementation-ready strategy for industrial-scale wastewater purification.

## Data Availability

All data generated or analyzed during this study are included in this article.
